# Supplementary material for: ZER1 Contributes to the Carcinogenic Activity of High-Risk HPV E7 Proteins
Source: mBio. 2022 Nov 8;13(6):e02033-22. doi: 10.1128/mbio.02033-22 (PMC9765665; doi:10.1128/mbio.02033-22)
Supplement: TABLE S1 [file mbio.02033-22-s0004.pdf]

Supplemental Table 1. Chimeric HPV16 and HPV18 E7 cloning

1. Chimera composition

HPV16 and HPV18 E7 Chimeras	Amino Acids
Chimera A	
CR1 CR2 HPV16 E7	1-39
C-terminus HPV18 E7	43-105
Chimera B	
CR1 CR2 HPV18 E7	1-42
C-terminus HPV16 E7	40-98
Chimera C	
CR1 HPV18 E7	1-15
CR2 C-terminus HPV16 E7	16-98

2. Primers for Chimeric HPV16 and HPV18 E7 Cloning

	Primer sequence 5' --> 3'
Chimera A	
HPV16 E7 GAW FWD	GGGGACAACTTTGTACAAAAAAGTTGGCaccATGCATGGAGATACACCTACATTG
CR1 CR2 HPV16 E7 REV	GTAAATGTTGATGATTAACTCCATCTATTTCATCCTCCTCCTCTGAG
C-terminus HPV18 E7 FWD	GAGGAGGAGGATGAAATAGATGGAGTTAATCATCAACATTTACCAGC
HPV18 E7 GAW REV	GGGGACAACTTTGTACAAGAAAGTTGGCTGCTGGGATGCACACCACGG
Chimera B	
HPV18 E7 GAW FWD	GGGGACAACTTTGTACAAAAAAGTTGGCaccATGCATGGACCTAAGGCAACATTG
CR1 CR2 HPV18 E7 REV	CTGCTTGTCCAGCTGGACCATCTATTTCATCGTTTTCTTCCTCTGAG
C-terminus HPV16 E7 FWD	GGAAGAAAACGATGAAATAGATGGTCCAGCTGGACAAGCAGAAC
HPV16 E7 GAW REV	GGGGACAACTTTGTACAAGAAAGTTGGTGGTTTCTGAGAACAGATGGG
Chimera C	
HPV18 E7 GAW FWD	GGGGACAACTTTGTACAAAAAAGTTGGCaccATGCATGGACCTAAGGCAACATTG
CR1 HPV18 E7 REV	GTAGAGATCAGTTGTCTCTGGTTGTAAATGCAATACAATGTCTTGC
CR2 C-terminus HPV16 E7 FWD	GACATTGTATTGCATTTACAACCAGAGACAACTGATCTCTAC
HPV16 E7 GAW REV	GGGGACAACTTTGTACAAGAAAGTTGGTGGTTTCTGAGAACAGATGGG

*GAW = Gateway recombinantion ATTB sites
Red = ATTB1 or ATTB2 sites
Black = E7 sequences
