# Supplementary material for: ZER1 Contributes to the Carcinogenic Activity of High-Risk HPV E7 Proteins
Source: mBio. 2022 Nov 8;13(6):e02033-22. doi: 10.1128/mbio.02033-22 (PMC9765665; doi:10.1128/mbio.02033-22)
Supplement: TABLE S2 [file mbio.02033-22-s0005.pdf]

Supplemental Table 2. Plasmids

Plasmid name	WhiteLab Plasmid #	Gene	Promoter	Bacterial resistance	Selectable marker	Tag	Tag Location
pDONR KozaK HPV16 E7	6487	HPV16 E7	N/A	Spectinomycin	N/A	N/A	N/A
pDONR Kozak HPV18 E7	6489	HPV18 E7	N/A	Spectinomycin	N/A	N/A	N/A
MSCV-neo C-HA GFP	8133	GFP	MSCV LTR	Ampicillin	Neomycin	HA	C-terminus
MSCV-neo C-HAonly HPV16 E7	6990	HPV16 E7	MSCV LTR	Ampicillin	Neomycin	HA	C-terminus
MSCV-neo C-HAonly HPV18 E7	6993	HPV18 E7	MSCV LTR	Ampicillin	Neomycin	HA	C-terminus
MSCV-neo C-HAonly HPV31 E7	6991	HPV31 E7	MSCV LTR	Ampicillin	Neomycin	HA	C-terminus
MSCV-neo C-HAonly ZER1	8382	ZER1	MSCV LTR	Ampicillin	Neomycin	HA	C-terminus
MSCV-neo C-HAonly HPV16 E7 E80A/D81A	8383	HPV16 E7	MSCV LTR	Ampicillin	Neomycin	HA	C-terminus
MSCV-IP N-HA Empty v2	7270	None	MSCV LTR	Ampicillin	Puromycin	N/A	N/A
MSCV-P C-FlagHA HPV16 E7	6640	HPV16 E7	MSCV LTR	Ampicillin	Puromycin	FlagHA	C-terminus
MSCV-P C-FlagHA HPV18 E7	6641	HPV18 E7	MSCV LTR	Ampicillin	Puromycin	FlagHA	C-terminus
MSCV-P C-FlagHA HPV16-18 E7 Chimera A	8235	HPV16-18 E7	MSCV LTR	Ampicillin	Puromycin	FlagHA	C-terminus
MSCV-P C-FlagHA HPV16-18 E7 Chimera B	8236	HPV16-18 E7	MSCV LTR	Ampicillin	Puromycin	FlagHA	C-terminus
MSCV-P C-FlagHA HPV16-18 E7 Chimera C	8237	HPV16-18 E7	MSCV LTR	Ampicillin	Puromycin	FlagHA	C-terminus
MSCV-P C-FlagHA HPV16 E7 Mutant 1	8304	HPV16 E7	MSCV LTR	Ampicillin	Puromycin	FlagHA	C-terminus
MSCV-P C-FlagHA HPV16 E7 Mutant 2	8305	HPV16 E7	MSCV LTR	Ampicillin	Puromycin	FlagHA	C-terminus
MSCV-P C-FlagHA HPV16 E7 H73A	8309	HPV16 E7	MSCV LTR	Ampicillin	Puromycin	FlagHA	C-terminus
MSCV-P C-FlagHA HPV16 E7 E80A/D81A	8310	HPV16 E7	MSCV LTR	Ampicillin	Puromycin	FlagHA	C-terminus
MSCV-P C-FlagHA HPV16 E7 ΔDLYC	7274	HPV16 E7	MSCV LTR	Ampicillin	Puromycin	FlagHA	C-terminus
MSCV-P C-FlagHA HPV18 E7 Q87E/Q88D	8384	HPV18 E7	MSCV LTR	Ampicillin	Puromycin	FlagHA	C-terminus

Plasmid name	WhiteLab Plasmid #	sgRNA sequence from Broad Brunello library	Promoter	Bacterial resistance	Selectable marker
LentiCRISPR v2 sgNT-1	8092	AGCTCGCCATGTCGGTTCTC	U6	Ampicillin	Puromycin
LentiCRISPR v2 sgZER1-1	8312	CAGGGACCCAATCAATCATG	U6	Ampicillin	Puromycin
LentiCRISPR v2 sgZER1-2	8313	AGGCTGAAGAAGCTCTCGTG	U6	Ampicillin	Puromycin
LentiCRISPR v2 sgPTPN14-3	8115	CCACACTGGACGTGAACGGG	U6	Ampicillin	Puromycin
LentiCRISPR v2 sgPTPN14-4	8116	TGTGCTTACCGTGTGAAAGA	U6	Ampicillin	Puromycin
