# Supplementary material for: ZER1 Contributes to the Carcinogenic Activity of High-Risk HPV E7 Proteins
Source: mBio. 2022 Nov 8;13(6):e02033-22. doi: 10.1128/mbio.02033-22 (PMC9765665; doi:10.1128/mbio.02033-22)
Supplement: TABLE S3 [file mbio.02033-22-s0006.pdf]

Supplemental Table 3. Antibody information

Antibodies	Company	Catalog number	Dilution
ZER1	GeneTex	GTX106983	1:1,000
Cullin 2	Bethyl	A302-476A	1:1,000
RB1	Calbiochem	OP66	1:500
Hypo-P RB1	BD Biosciences	554164	1:500
HA-Peroxidase	Roche	12013819001	1:500
GAPDH	Invitrogen	MA5-15738	1:5,000
ACTIN	Millipore	MAB1501	1:10,000
B-ACTIN	Cell Signaling Technology	3700S	1:1,000
PTPN14	Cell Signaling Technology	13808S	1:500
Anti-Mouse HRP-linked	Cell Signaling Technology	7076S	1:2,000
Anti-Rabit HRP-linked	Cell Signaling Technology	7074S	1:2,000
